# Spontaneous Pneumothorax: A Tale of Two Unique Cases

**DOI:** 10.7759/cureus.32544

**Published:** 2022-12-15

**Authors:** Marc Assaad, Khalil El Gharib, Hussein Rabah, Ali Kassem, Ahmad Abou Yassine, Loai Dahabra, Manuel Villa Sanchez, Thomas Kilkenny

**Affiliations:** 1 Internal Medicine, Staten Island University Hospital, New York City, USA; 2 Internal Medicine, Northwell Health, Staten Island, USA; 3 Thoracic Surgery, Staten Island University Hospital, New York City, USA; 4 Pulmonary and Critical Care Medicine, Staten Island University Hospital, New York City, USA

**Keywords:** pulmonary bleb, pleurectomy, pleurodesis, thoracoscopy, pneumothorax, chronic obstructive lung disease, emphysema

## Abstract

We herein present two cases of spontaneous pneumothorax. The first one is occurring in an elderly female who has an extensive history of smoking and an underlying chronic obstructive lung disease, whereas the second case represents a congenital bleb in a male patient who has no other underlying pulmonary disorder. Both cases presented to our facility with a spontaneous pneumothorax following pulmonary bleb rupture. Both patients underwent thoracoscopic surgery with subsequent partial pleurectomy and pleurodesis.

## Introduction

Pneumothorax results from the accumulation of air in the pleural space that resides between the chest wall and the lung, leading to partial or complete lung collapse. This complication occurs more frequently in males than in females [1]. Pneumothorax is categorized as spontaneous or traumatic [2]. Spontaneous pneumothorax (SP) is in its turn divided into primary spontaneous pneumothorax (PSP) and secondary spontaneous pneumothorax (SSP), which occur in the absence or presence of an underlying pulmonary disease, respectively. In PSP, despite the absence of lung disease, underlying blebs or bullae are identified. Risk factors include tall and thin stature, smoking, and change in altitude. On the other hand, SSP, which is the least common among SP, occurs in patient with lung disease, such as chronic obstructive pulmonary disease (COPD) [2,3]. Symptoms vary from asymptomatic to severe hemodynamic collapse such as represented in tension pneumothorax. Presentation is quite often related to respiratory symptoms including difficulty breathing, chest pain, and tachypnea [1,4,5]. We herein report two cases of SP presenting to our facility complaining of chest pain and dyspnea and who underwent surgical approach.

## Case presentation

Case 1

A 75-year-old female, known to have systemic essential hypertension, diverticulosis resulting priorly in lower gastrointestinal bleed, aortic valve replacement on coumadin, dyslipidemia, carotid artery stenosis treated with endarterectomy, and hypothyroidism, presented to the emergency department complaining of worsening dyspnea. The patient has a smoking history of 60 pack-year. She was diagnosed with chronic obstructive pulmonary disease (COPD), for which she is being treated with bronchodilators, needless of supplemental oxygen. The patient has severe emphysematous lungs when seen on computed tomography (CT) of the chest. The history begins six months prior to presentation, when the patient sustained a mechanical fall at home, for which she was not evaluated. Since the fall, the patient has been complaining of shortness of breath on exertion, without response to her previous regimen of bronchodilators. The shortness of breath has worsened in recent weeks when its maximal tolerated activity is estimated at 20 feet. The patient denies fever, chills, new phlegm, hemoptysis, lower extremity swelling, weight, or appetite changes. On admission, the patient was tachypneic with a respiratory rate of 25 breaths per minute, oxygen saturation of 88% on room air, and with a heart rate of 125 beats per minute. The patient was afebrile and normotensive at 114/57 mmHg. On examination, the patient was alert, in mild respiratory distress, with accessory muscle use, and was following commands. Physical examination did not reveal any jugular venous distention, lower extremity swelling, crackles, or wheezes. Lung examination was relevant for diminished breath sounds, promptly on the right side, with ipsilateral hyperresonance to percussion. Initial blood work showed normal red and white blood cell count along with normal kidney function and electrolytes. There were no acute ischemic changes on the electrocardiogram.

**Figure 1:**
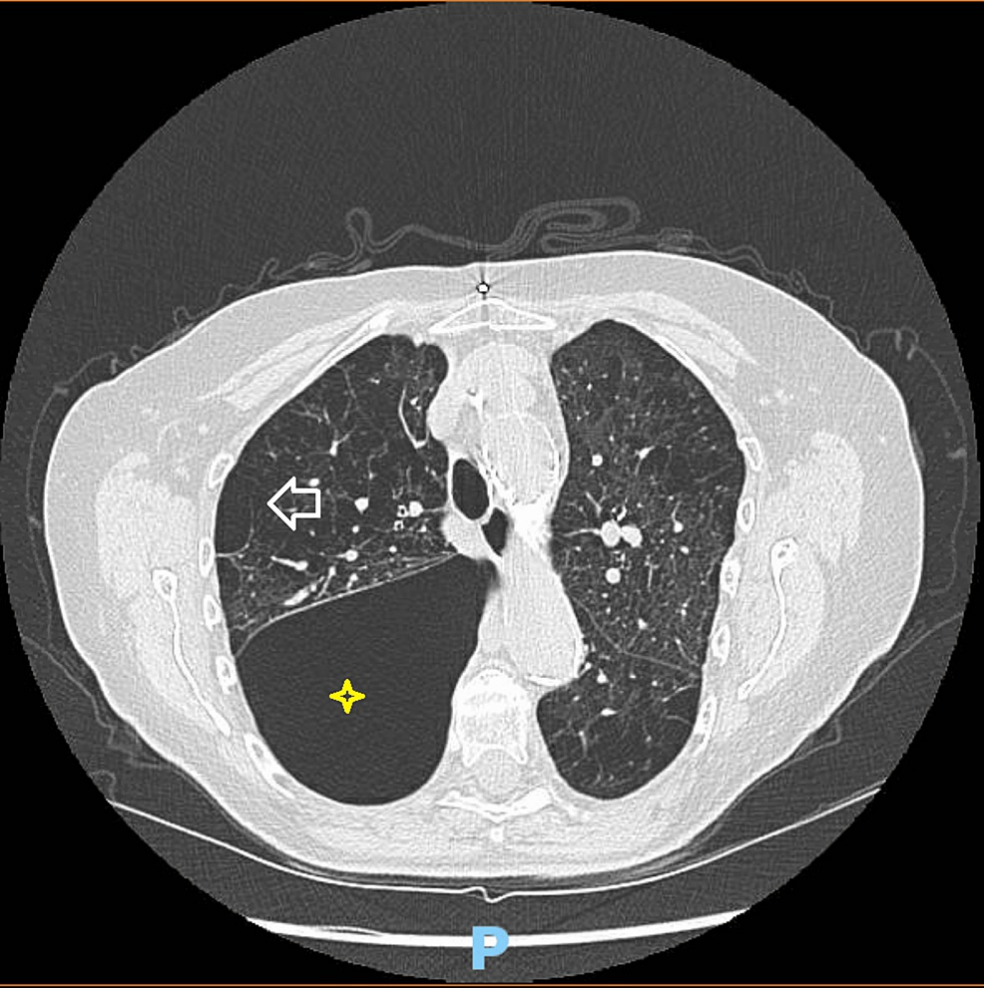
CT chest showing a pneumothorax (asterisk) measuring 11.9 x 8.9 cm with extensive emphysema (arrow) of the bilateral lung parenchyma.

In this setting, an urgent chest x-ray (CXR) was obtained, revealing a large right-sided pneumothorax. This test was completed by a CT of the chest which showed an air collection of 11.9 x 8.9 cm within the posterior right lung field compatible with pneumothorax, along with a large bleb amongst the emphysematous changes. There was no evidence of tension pneumothorax (Figure 1). The patient was placed on supplemental oxygen through nasal cannula, and her oxygen saturation was maintained at 95%. An urgent 28 French chest tube was placed on the right side by the cardiothoracic team. A continuous air leak was present from the chest tube drain. The following day the patient underwent video-assisted thoracoscopic surgery (VATS) when the lung did not fully inflate after the interventions. A large bleb was seen in the right upper lobe and successfully resected. It was hypothesized that the bleb ruptured after the fall as this is when the dyspnea began. A partial pleurectomy was additionally performed along with talc pleurodesis.

**Figure 2:**
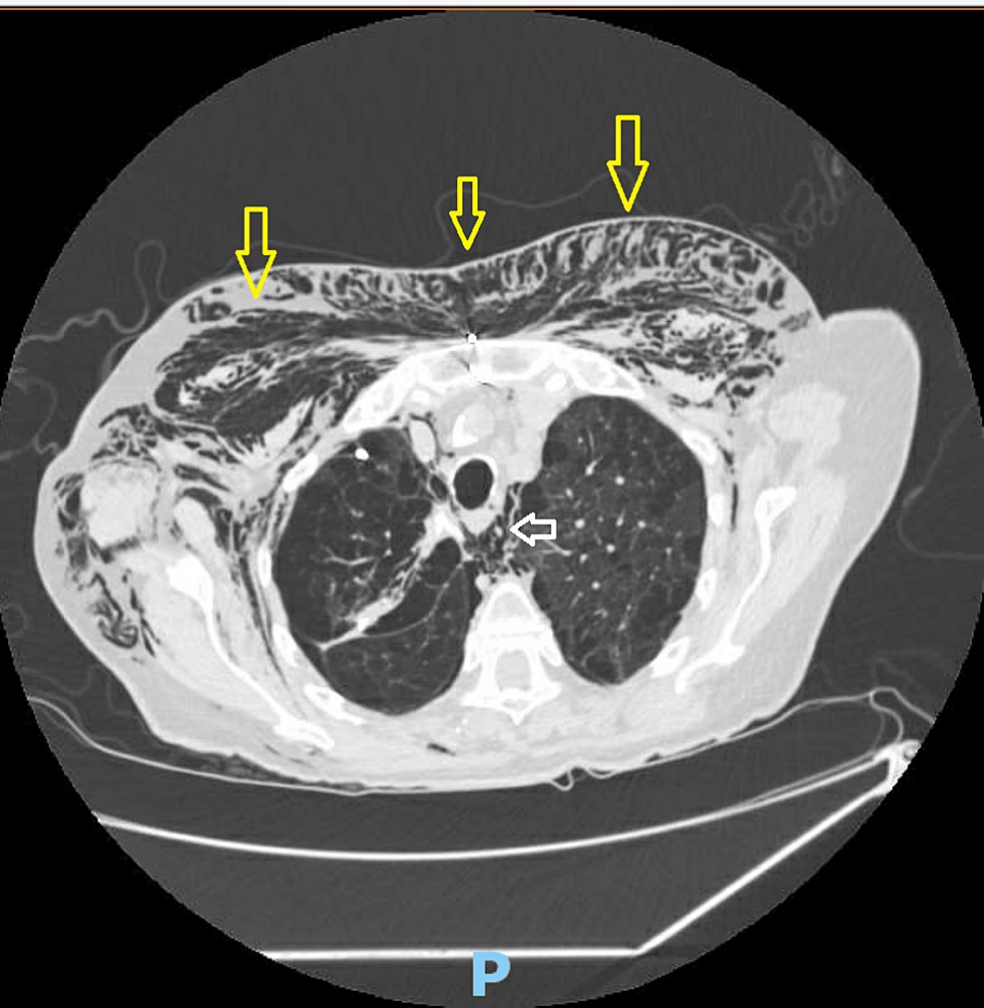
CT chest showing resolution of the pneumothorax. The image shows new pneumomediastinum (white arrow) and extensive subcutaneous emphysema involving the chest wall (yellow arrows).

A follow-up CT chest was performed on day eight post operatively showing resolution of the pneumothorax, but extensive subcutaneous emphysema of the chest wall and pneumomediastinum were newly reported (Figure 2). Surgical chest tube was removed on day nine post procedure and the remaining pigtail was removed on day 12. The patient was safely discharged to a rehabilitation facility after being hospitalized for two weeks. Her functional status has improved with no aggravation of her respiratory status and no recurrence of the pneumothorax.

Case 2

A 59-year-old male with type 2 diabetes mellitus, who has no underlying lung disease, presented to our facility for acute onset of chest pain and shortness of breath. The patient is an active smoker with a history of smoking 20 pack-year. Upon admission, patient was hemodynamically stable, without hypoxemia, and was requiring supplemental oxygenation through nasal cannula only for comfort. Physical examination was remarkable for mild respiratory distress, tachypnea with a rate of 22 breaths per minute and tachycardia. Breath sounds were diminished on the left side of the chest. Otherwise, physical findings were unremarkable as well as laboratory results. Viewing the respiratory complaints, an urgent CXR revealed a left-sided pneumothorax with air gap measuring 7.6 cm without mediastinal shift. A small-bore chest tube was inserted with mild relief of patient’s symptomatology. Incentive spirometry was provided. Supplemental oxygenation via a nasal cannula was also provided.

**Figure 3:**
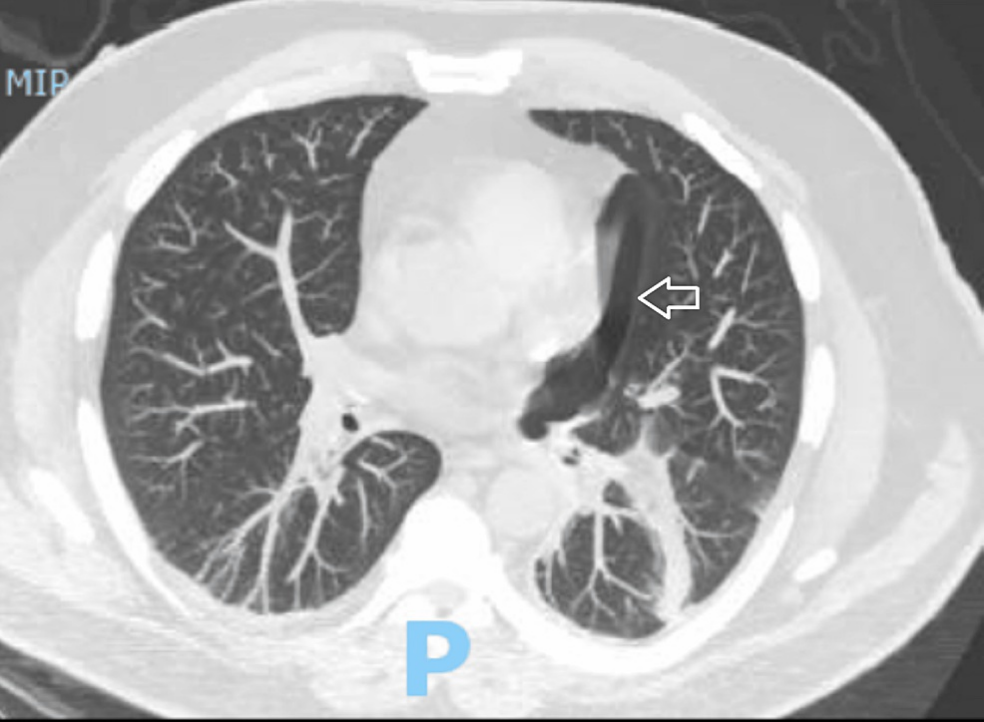
Chest x-ray showing left pneumothorax (arrow) with air gap measuring 7.6 cm.

**Figure 4:**
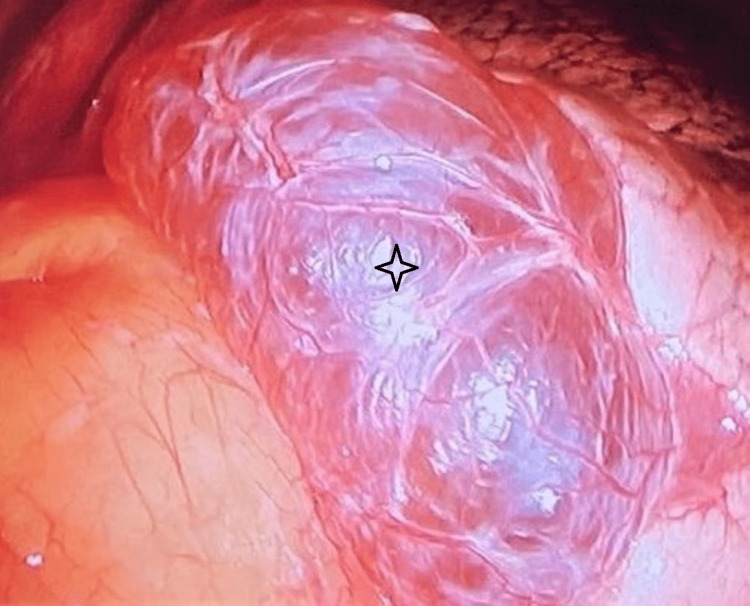
Thoracoscopic image showing the pleural bullae measuring 10 cm (asterisk).

A follow-up CXR on the next day was notable for the persistent pneumothorax. A CT scan of the chest was performed showing left lower lobe medial 10.2 cm pleural bleb (Figure 3). Cardiothoracic surgical team was consulted and recommended VATS, as patient was an exemplary candidate. The patient underwent subsequent bullectomy with partial pleurectomy and chemical pleurodesis (Figure 4). Having a single bulla with a size of almost 10 cm occurring medially in the lung equivoques congenital or primary bleb. Patient was diagnosed with SSP and was discharged within two weeks of admission. The patient was examined by the pulmonary team in the clinic two weeks after his initial discharge, then three months thereafter with no recurrence of his symptoms.

## Discussion

In this report, we illustrated the case of an elderly female presenting to our facility with SSP due to a large emphysematous bleb who underwent surgical repair with VATS. The patient was successfully managed and discharged two weeks after her presentation with complete resolution of her pneumothorax.

SSP is less common than PSP [6] and usually is more symptomatic than PSP upon presentation due to concomitant underlying pulmonary disorder; SSP has both higher mortality and recurrence rate [7]. COPD is the most common etiology behind SSP, whereas tuberculosis is the most common in the endemic areas [5,6]. Spontaneous pneumothorax has bimodal age presentation - PSP presents in younger population and SSP tends to occur in elderly population with concomitant comorbidities [6]. Short-term complications are subcutaneous emphysema, infections, and death. The long-term complication is mainly recurrences [8]. To decrease the recurrence rate, bullectomy and chemical or surgical pleurodesis are performed when appropriate [9].

Therapeutic approaches vary accordingly. The most common path is the placement of chest tube when the pneumothorax size is greater or equal to 3 cm according to the American College of Chest Physicians, or resultant of hemodynamic instability [5]. Small and large bore chest tubes have the same efficacy in drainage [2,6]. Failure to yield complete resolution of the pneumothorax by day three to five should prompt surgical intervention and thoracoscopy or VATS has largely replaced open thoracotomy in this particular setting [5]. Data suggest lower recurrence rate and shorter hospitalization for VATS as compared to open surgical techniques [7] whereas other meta-analysis offers opposite findings [9]. Other therapeutic modalities include bronchial occlusion, valve insertion [7,10], or lung transplant if indicated in the context of underlying severe lung disease [6].

Lung volume reduction surgery (LVRS) may be indicated when elevated residual volume and hyperinflation occur, and the diffusion lung capacity of carbon oxide (DLCO) remains acceptable for operability [11]. Endoscopic techniques have also been effective in improving spirometry parameters in patients with emphysema [12].

A comparison study was conducted by Petro et al., comparing the pulmonary outcome of patients with a localized bulla with those who had diffuse emphysematous changes after surgical intervention (bullectomy). Patients with single bullae had better respiratory outcome post operatively. Some indications were established which include the size of the bullae and patient’s symptomatology [13]. A major limitation of this study is the sample size. More randomized clinical trials and long-term follow up are needed. However, as for prevention, there is no consensus for the management and follow-up of bullous changes, but it may be beneficial to identify patients with bullae who are at high risk of SSP development and preventive LVRS, although possibly successful, remains questionable in this circumstance.

## Conclusions

Spontaneous pneumothorax is a complication that arises in patients with emphysematous lungs or pulmonary blebs. No clear guidelines impose monitoring or intervention to prevent pneumothorax from occurring. Patients with large blebs should be aware of the risk of rupture and of the clinical presentation of this complication. Surgical resection of the bleb might be beneficial in patients at high risk of pneumothorax.
